# Outcome selection, measurement and reporting for new surgical procedures and devices: a systematic review of IDEAL/IDEAL‐D studies to inform development of a core outcome set

**DOI:** 10.1002/bjs5.50358

**Published:** 2020-10-04

**Authors:** R. C. Macefield, N. Wilson, C. Hoffmann, J. M. Blazeby, A. G. K. McNair, K. N. L. Avery, S. Potter

**Affiliations:** ^1^ National Institute for Health Research Bristol Biomedical Research Centre, Bristol Centre for Surgical Research, Bristol Medical School University of Bristol Bristol UK; ^2^ Department of Gastrointestinal Surgery Bristol UK; ^3^ Bristol Breast Care Centre North Bristol NHS Trust Bristol UK

## Abstract

**Background:**

Outcome selection, measurement and reporting for the evaluation of new surgical procedures and devices is inconsistent and lacks standardization. A core outcome set may promote the safe and transparent evaluation of surgical innovations. This systematic review examined outcome selection, measurement and reporting in studies conducted within the IDEAL (Idea, Development, Exploration, Assessment and Long‐term monitoring) framework to examine current practice and inform the development of a core outcome set for early‐phase studies of surgical procedures/devices.

**Methods:**

Web of Science and Scopus citation searches were performed to identify author‐reported IDEAL/IDEAL‐D studies for any surgical procedure/device. Outcomes were extracted verbatim, including contextual information regarding outcome selection and measurement. Outcomes were categorized to inform a conceptual framework of outcome domains relevant to evaluating innovation.

**Results:**

Some 48 studies were identified. Outcome selection, measurement and reporting varied widely across studies in different IDEAL stages. From 1737 outcomes extracted, 22 domains specific to evaluating innovation were conceptualized under seven broad categories: procedure completion success/failure; modifications; unanticipated events; surgeons' experiences; patients' experiences; resource use specific to the innovative procedure/device; and other innovation‐specific outcomes. Most innovation‐specific outcomes were measured and reported in only a small number of studies.

**Conclusion:**

This review highlighted the need for guidance and standardization in outcome selection and reporting in the evaluation of new surgical procedures/devices. Novel outcome domains specific to innovation have been identified to establish a core outcome set for future evaluations of surgical innovations.

## Graphical abstract

This review of IDEAL/IDEAL‐D studies examined outcome selection, measurement and reporting to examine current practice and identify outcome domains unique to evaluating innovation. The findings have informed the development of a core set of outcome domains to measure and report in all early studies of innovative surgical procedures/devices to promote standardized, safe and transparent evaluation.

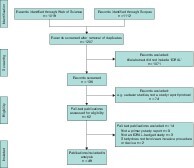

Potential domains identified

## Abstract (es)

**Antecedentes:**

La selección de resultados, mediciones y redacción de los informes para la evaluación de nuevos procedimientos y dispositivos quirúrgicos es inconsistente y carece de estandarización. Determinar un conjunto de resultados básicos (*core outcome set*, COS) podría contribuir a la transparencia y seguridad de las evaluaciones de las innovaciones quirúrgicas. Esta revisión sistemática analizó la selección de resultados, medición de los mismos e informes de estudios efectuados en el marco metodológico IDEAL, a fin de valorar la práctica actual y presentar el desarrollo de un COS para estudios en fase inicial de procedimientos/dispositivos quirúrgicos.

**Métodos:**

Se realizaron búsquedas en las bases de datos Web of Science y Scopus para identificar estudios efectuados por los autores en el marco IDEAL/IDEAL‐D referentes a cualquier procedimiento/dispositivo quirúrgico. Los resultados se extrajeron literalmente, incluida la información contextualizada referente a la selección y medición de los resultados. Los resultados se categorizaron para presentar información en un marco conceptual de dominios de resultados que fueran relevantes para poder evaluar la innovación.

**Resultados:**

Se identificaron 48 estudios. Los estudios mostraban una amplia variedad en la selección de resultados, mediciones e información correspondientes a diferentes estadios IDEAL. A partir de 1.737 resultados extraídos, se conceptualizaron 22 dominios específicos para evaluar la innovación agrupados en 7 amplias categorías: éxito/fracaso para completar el procedimiento; modificaciones; eventos imprevistos; experiencias de los cirujanos; experiencias de los pacientes; uso de recursos específicos del procedimiento/dispositivo innovador y otros resultados específicos de la innovación. La mayoría de los resultados específicos de la innovación se midieron y notificaron solo en un pequeño número de estudios.

**Conclusión:**

Esta revisión ha puesto de manifiesto la necesidad de orientación y estandarización en la selección de resultados y la notificación en la evaluación de nuevos procedimientos/dispositivos quirúrgicos. Se han identificado nuevos dominios de resultados específicos de innovación para establecer un COS para futuras evaluaciones de innovaciones quirúrgicas.

## Introduction

Unlike pharmaceuticals, where the introduction and evaluation of new medicines into clinical practice is highly regulated, the process for introducing new procedures and devices in surgery is inconsistent and lacks standardization. This has resulted in several high‐profile cases of potentially harmful interventions becoming established in clinical practice without robust evaluation, including vaginal mesh and metal‐on‐metal hip implants^1^, ^2^.

A key problem in the effective evaluation of new surgical procedures and devices is the lack of standardization in outcome selection, measurement and reporting. Studies may measure benefits and harms in different ways, so that results cannot be directly compared or combined. Furthermore, investigators can choose which outcomes to measure and report, introducing bias. There is a need for standardized and transparent measurement and reporting of outcomes to improve safe and efficient evaluation of surgical innovation for introduction into clinical practice.

The development and use of a core outcome set (COS) – a minimum set of outcome domains to be measured and reported in all early‐phase evaluations of innovative surgical procedures and devices – may be a solution to addressing inconsistent and heterogeneous outcome selection and reporting^3^. The methodology for the development of a COS in effectiveness studies is well established, including taxonomies to classify outcomes^4^, ^5^. However, standard methods to identify outcomes relevant to later‐phase effectiveness studies, when the intervention under evaluation is no longer being modified, may not identify outcomes specifically relevant to evaluating surgical innovation. Data sources that specifically focus on innovation are required to provide insight into outcome selection and identify potentially relevant and meaningful outcomes for the first step in developing a COS in this setting.

The IDEAL (Idea, Development, Exploration, Assessment and Long‐term monitoring) framework describes a pathway for evaluating surgical innovations from first‐in‐man through to long‐term evaluation^6^. The framework has also recently been adapted for evaluation of new medical devices (IDEAL‐D)^7^. It is hypothesized that investigators who have engaged with the IDEAL framework when designing and conducting studies may have considered outcome selection and reporting specifically in the context of evaluation of surgical innovation. As such, these studies may provide significant insight into outcomes that have particular relevance to the process of innovation, offering a valuable data source to identify outcomes and contribute to the conceptualization of outcome domains to inform the development of a COS for early‐phase studies.

This study aimed to examine outcome selection, measurement and reporting in author‐reported IDEAL/IDEAL‐D studies to identify current practice, and serve as one of multiple data sources to help conceptualize novel domains relevant to evaluating innovation to inform the development of a COS^8^.

## Methods

A systematic review and content analysis of author‐reported IDEAL/IDEAL‐D studies was undertaken following PRISMA guidelines^9^, where applicable. This review is one of multiple data sources used to inform the wider study to develop a COS for studies of surgical innovation. A detailed protocol for this systematic review was not registered specifically as methods have been included in the COS development protocol, which has been published previously^8^.

### Identification of studies

Electronic searches were performed to identify studies reported as following the IDEAL/IDEAL‐D framework. Searches were undertaken in databases with citation tools (Web of Science and Scopus) to identify all publications citing any of ten key IDEAL/IDEAL‐D papers^6^, ^7^, ^10^, ^11^, ^12^, ^13^, ^14^, ^15^, ^16^, ^17^ deemed significant in describing the framework by members of the IDEAL collaboration^18^. Searches were carried out in April 2019. No restrictions to study design or publication dates were applied. Search results were imported into reference management software and duplicates removed. Results were filtered to select and retain records with the text word IDEAL appearing in the title or abstract.

### Selection of studies

Titles and abstracts were screened independently for eligibility by two reviewers. Reports or protocols for studies described as IDEAL or IDEAL‐D in the title or abstract were included. Eligible studies were: studies of innovative invasive procedures; studies of innovative devices; or studies where the innovation was something other than an invasive procedure/device but the study involved an invasive procedure, for example radiological imaging for guided biopsy. The latter category of studies was included owing to the potential value of such studies for identifying outcomes relevant to evaluating innovation. Invasive procedures were defined using an existing published definition and included ‘purposeful or deliberate access to the body gained via an incision, percutaneous puncture, where instrumentation is used in addition to the puncture needle, or instrumentation via a natural orifice’^19^. Systematic reviews, book chapters, secondary studies and studies that did not involve living human participants (such as cadaver, animal or simulation studies) were excluded. Studies using adaptions of the IDEAL framework that did not include or involve an invasive procedure or device (such as non‐surgical complex interventions) were also excluded, along with letters, commentaries, editorials and conference proceedings. Screening results were compared for consistency and discrepancies resolved by discussion. Full‐text copies of potentially relevant publications were obtained and checked for eligibility. Reasons for excluding publications for which full‐text copies had been obtained were recorded. Publications of uncertain relevance were discussed within the study team.

### Data extraction

A standardized electronic data extraction form (REDCap software^20^) was developed and piloted by the study team. Data extraction was performed by one reviewer with a second reviewer checking 10 per cent of publications for accuracy and completeness. Discrepancies were resolved by discussion and any additional verbatim outcomes identified during this process were extracted.

Study characteristics were extracted including: year of publication, details of the innovation, author‐reported IDEAL stage, number of patients, participating centres and surgeons, and geographical origin of the study. All measured and/or reported outcomes were extracted verbatim together with any contextual information regarding the selection and measurement of outcomes. Rationale for outcome selection was categorized as: detailed, with a hypothesized effect (for example, anticipated improvement in at least one outcome); detailed, but with no hypothesized effect for any outcomes; general rationale (for example ‘to examine safety’); or no rationale provided^21^. Contextual information considered potentially relevant to evaluating the process of innovation was extracted, where applicable. This included verbatim text relating to: stopping the innovation or making changes to the procedure/device in future use or application; limitations in study interpretation or conclusion in relation to outcomes; outcome assessment in future studies; and assessment of the surgical learning curve^22^. In studies where modifications to the procedure or patients selected to undergo the procedure were reported, details of how these were reported (for example, text, graphs or tables) were extracted. Data were recorded in a specifically designed study database^20^.

### Data analysis

Data analysis was undertaken in two stages. Extracted verbatim outcomes were categorized individually into domains to develop a preliminary framework of outcome domains relevant to innovation. Categorization was performed by one reviewer. During this process, the conceptual framework was modified iteratively until data saturation was reached and no new outcome domains were identified (all outcomes had been categorized). A provisional conceptual framework of domains was subsequently attained. A subsample of outcomes (81, from 2 publications selected at random) were independently categorized by a second reviewer with clinical expertise to ensure methodological rigour. Discrepancies were resolved by discussion with the wider study team.

In the second stage of analysis, all outcomes were recategorized using the provisional conceptual framework by a second independent reviewer. Source documents (IDEAL/IDEAL‐D study publications) were referred to for context, for example, to determine whether clinical outcomes were anticipated or unanticipated. Outcomes that were not specific enough to categorize (for example, generic descriptions such as safety or feasibility) were coded as ‘too broad for categorization’. Outcomes that did not specifically address the evaluation of the innovation but were routinely measured data variables of interest to the clinical specialty were coded as common data elements^23^.

Outcome domains within the provisional conceptual framework were further classified by the authors as being innovation‐specific or shared with effectiveness studies. Innovation‐specific domains included non‐traditional outcome domains (different to outcome domains typically measured in later‐phase studies) considered to be conceptually specific to evaluating the ongoing process of innovation when the procedure/device was new or evolving. Examples included modifications to the surgical procedure/device and whether the procedure was completed successfully. Domains shared with effectiveness studies encompassed outcomes that would commonly be measured in later‐phase studies^4^ considered not unique to evaluating innovation. Examples included mortality and patient‐reported outcomes such as postoperative pain and physical function.

Study characteristics, measured/reported outcomes (based on the derived conceptual framework of domains) and contextual information relevant to selecting or measuring outcomes or evaluating innovation were compared across IDEAL stages specifically reported by the authors. Descriptive statistics were summarized using Microsoft Excel® (Microsoft, Redmond, Washington, USA) and STATA® statistical software (StataCorp, College Station, Texas, USA).

Assessment of factors relevant to systematic reviews of effectiveness (such as study quality or risk of bias) and data syntheses (for example meta‐analyses of outcome data) were not appropriate given the exploratory aims of this review. Findings were synthesized and tabulated using descriptive statistics and a narrative summary directed at the study aims.

**Fig. 1:**
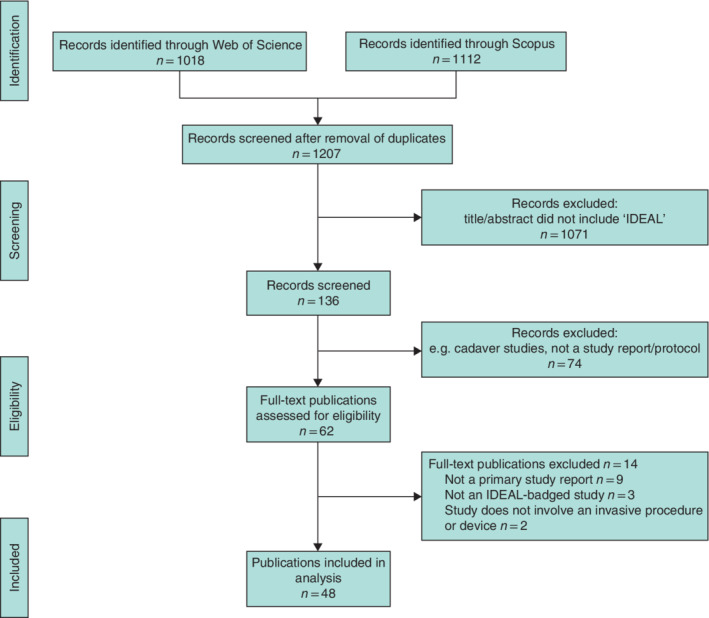
PRISMA diagram showing selection of articles for review

## Results

After removal of duplicates, the search yielded 1207 records citing any of the ten key IDEAL/IDEAL‐D publications. Of these, 136 (11·3 per cent) included IDEAL as a text word in the title or abstract. Initial screening identified 62 of 136 records (45·6 per cent) for full‐text eligibility assessment. Finally, 48 relevant publications (42 study reports, 6 protocols) were included in the analysis^24^, ^25^, ^26^, ^27^, ^28^, ^29^, ^30^, ^31^, ^32^, ^33^, ^34^, ^35^, ^36^, ^37^, ^38^, ^39^, ^40^, ^41^, ^42^, ^43^, ^44^, ^45^, ^46^, ^47^, ^48^, ^49^, ^50^, ^51^, ^52^, ^53^, ^54^, ^55^, ^56^, ^57^, ^58^, ^59^, ^60^, ^61^, ^62^, ^63^, ^64^, ^65^, ^66^, ^67^, ^68^, ^69^, ^70^, ^71^ (*Fig*. *1*).

Included publications are detailed in *Table* *1*. The majority were published in 2017 or 2018 (14 and 13 respectively). Thirty‐nine publications were from Europe. Twenty‐four described studies of innovations in surgical procedures and ten described studies of four different new devices. The remaining 14 publications described non‐surgical innovations that included a surgical procedure. The specific IDEAL stage(s) describing the evaluative stage of the innovation was generally reported. Two publications, however, did not specify the IDEAL stage. Frequencies of author‐reported IDEAL stages were: stage 1 (Idea, 11); stage 2a (Development, 16); stage 2b (Exploration, 9); stage 3 (Assessment, 1); and multiple stages (9).

**Table 1:** Characteristics of included publications

	No. of publications (*n* = 48)
**Type of publication**	
Study report	42
Protocol	6
**Year of publication**	
2019	1
2018	13
2017	14
2016	6
2015	4
2014	5
2013	0
2012	2
2011	3
**Country of study**	
Europe (non‐UK)	33
Asia	8
UK	6
North America	1
**Type of innovation**	
Innovative procedure	24
Device	10
Other innovation that involved surgery*	14
**Author‐reported IDEAL stage**	
1	11
2a	16
2b	9
3	1
Multiple stages	9
Not stated	2

* Such as radiological imaging.

### Characteristics of IDEAL/IDEAL‐D studies by stage

Characteristics of studies across the different IDEAL stages (stated in 46) are summarized in *Table* *2*. The number of centres involved, number of surgeons/operators and number of included patients were broadly in line with the recommendations of the IDEAL framework. For example, early‐phase studies (such as IDEAL stages 1 and 2a) typically involved one or two centres, one or two surgeons and a small number of patients, with numbers increasing in stage 2b and stage 3 studies. The exception was one IDEAL‐D stage 1 study^50^ describing the use of a type of mesh for cystocele repair that included 37 women. The median number of outcomes per study was relatively similar across IDEAL stages 1, 2a and 2b (median 32, 38 and 36 respectively). The range in number of outcomes described per study, however, varied considerably. For example, in IDEAL stage 1 studies, the number of outcomes described per study ranged from six to 82.

**Table 2:** Comparison of study characteristics, outcome selection and reporting in publications by IDEAL stage (*n* = 46)

	IDEAL stage				
	1 (*n* = 11)	2a (*n* = 16)	2b (*n* = 9)	3 (*n* = 1)	Multistage (*n* = 9)
**Study characteristics**					
No. of centres					
Single centre	11	15	6	0	6
Multicentre (2 centres)	0	1	0	1	1
Multicentre (> 2 centres)	0	0	2	0	1
Not reported	0	0	1	0	1
No. of surgeons/operators					
1	4	6	1	0	1
2	1	2	0	0	1
> 2	0	1	2	1	4
Not reported/unclear	6	7	6	0	3
No. of included patients					
< 10	7	0	0	0	0
10–20	3	11	1	0	2
21–50	1	4	1	0	3
> 50	0	1	7	1	4
Not reported	0	0	0	0	0
No. of verbatim outcomes per study*	32 (6–82)	38 (15–56)	36 (11–74)	17 (17)	30 (10–63)
**Reported context for the selection and measurementof outcomes**					
Rationale for outcome selection					
Detailed, with hypothesized effect for at least one outcome	3	4	4	0	1
Detailed, but with no hypothesized effect for any outcomes	2	2	0	0	1
General rationale	4	6	4	1	3
No rationale provided	2	4	1	0	4
**Reported context relating to outcomes and evaluatinginnovation**					
Text relating to stopping the innovation/making changes to the procedure/device in future applications of use†	5	6	0	n.a.	4
Reported limitations in interpretation/conclusions in relation to outcomes†	5	4	4	n.a.	5
Outcome assessment in future studies†	9	9	5	n.a.	6
Any mention of a learning curve	4	8	5	0	2

* Values are median (range). The analysis includes 46 studies where the authors stated an IDEAL stage.

† Excluding protocols. n.a., Not applicable (protocol).

### Outcome selection, measurement and reporting in IDEAL/IDEAL‐D studies

Most studies (37 of 48) provided a rationale for outcome selection. Often (20 studies) this was a general rationale (for example ‘to examine safety’). Fewer studies (12) provided detail on the expected or anticipated effect on outcomes. Instead, outcomes were reported to be measured without explanation or elaboration regarding their selection. There were no observed differences in the level of detail provided in the rationale between studies of different IDEAL stages (*Table* *2*). Studies that mentioned stopping the innovation or making changes to the procedure/device in future applications of its use were broadly in line with the recommendations of the IDEAL framework. For example, 15 of 42 study reports (excluding protocols) included text to this effect, most of these being IDEAL stage 1 or 2a studies where innovations are less stable.

Limitations to the interpretation of findings or study conclusions in relation to outcomes were discussed in 18 of 42 study reports. These included, for example, the short follow‐up time for measuring outcomes and being underpowered to detect potential adverse events with a low incidence. Recommendations/suggestions for outcome assessment in subsequent future studies were discussed in 30 of 42 study reports. These were primarily about generic outcomes, such as measuring efficacy and safety. Four studies provided specific recommendations for outcome assessment in future studies, including two that recommended the inclusion of patient‐reported outcome measures. Fewer than half of the studies (20 of 48) mentioned the learning curve. The frequency of mentioning the learning curve did not differ by IDEAL stage.

### Types of outcome

A total of 1737 outcomes were extracted from the 48 studies and categorized into outcome domains. The derived conceptual framework comprised 32 domains, 22 of which were considered to be conceptually specific to evaluating innovation and ten to be shared with effectiveness studies (*Table* *3*). The majority of outcomes (1098, 63·2 per cent) were categorized into domains shared with effectiveness studies (*Table S1*, supporting information), for example, outcomes assessing whether the overall desired effect of the procedure/device had been achieved, such as the number of positive surgical margins in a study of a new surgical procedure in oncology^28^. Other outcome domains shared with effectiveness studies included anticipated disadvantages, such as adverse events and complications, duration of the procedure, duration of hospital stay, and patient's physical/psychological experiences after the procedure. A smaller number of outcomes (552, 31·8 per cent) were categorized into the 22 domains considered to be conceptually specific to evaluating innovation. These are described in detail below under seven broad subheadings.

**Table 3:** Conceptual framework of outcome domains

Broad classification	Domain number	Outcome domain
**Innovation‐specific domains**	1–2	Procedure completion success/failure
	3–6	Modifications: to the procedure; to concomitant interventions; and to patient selection during study
	7–9	Unanticipated advantages: during the procedure; after the procedure – short term; and after the procedure – long term
	10–12	Unanticipated disadvantages: during the procedure; after the procedure – short term; and after the procedure – long term
	13	Surgeon/operator's experience of the innovative procedure/device
	14–15	Patient's experience of the innovative procedure/device, including: physical experiences during procedure, if applicable; and psychological experience of having the innovative procedure/device
	16–19	Required resource use specific to the innovative procedure: before the procedure; during the procedure; after the procedure during the hospital stay; and after leaving hospital
	20	Details of patients suitable for the procedure in future
	21	Details of operator training/expertise necessary to perform the procedure in future
	22	Mechanical/technical problems with device, if applicable
**Domains shared with effectiveness studies**	23	Overall desired effect of procedure/device achieved
	24–26	Anticipated advantages: during the procedure; after the procedure – short term; and after the procedure – long term
	27–29	Anticipated disadvantages: during the procedure; after the procedure – short term; and after the procedure – long term
	20	Duration of procedure
	31	Duration of hospital stay
	32	Patient's physical/psychological experiences after the procedure

### Innovation‐specific outcome domains

#### Procedure completion success/failure

Some 107 outcomes (6·2 per cent) were categorized as relating to the success or failure of performing the innovative procedure or using the innovative device (*Table S1*, supporting information). Examples of success included outcomes assessing whether all the steps of performing the innovative procedure were completed as planned. Examples of failure included the number of patients for which the planned innovative procedure was abandoned or changed to an alternative procedure. Procedure completion success/failure outcomes were measured or reported in most studies (33 of 48) (*Table* *4*). Relative consistency was observed in the proportion of studies reporting success/failure outcomes across the IDEAL stages.

**Table 4:** Innovation‐specific outcomes measured/reported in studies by IDEAL stage (n = 46)*

	IDEAL stage				
	1 (*n* = 11)	2a (*n* = 16)	2b (*n* = 9)	3 (*n* = 1)	Multistage (*n* = 9)
Outcomes relating to the success or failure of performing the procedure/ using the device	8	12	5	1	7
Outcomes relating to modifications	5	11	3	0	5
Outcomes relating to unanticipated event (advantages/disadvantages)	1	5	0	0	2
Outcomes relating to surgeon/operator experience	5	8	3	0	7
Outcomes relating to patient experience	1	4	1	0	0
Outcomes relating to resource use	9	9	6	0	7

The analysis includes 46 studies where the authors stated an IDEAL stage.

#### Modifications

Some 92 outcomes (5·3 per cent) were categorized as relating to modifications. This included modifications to the individual steps of the planned innovative procedure (for example, a technical refinement to the procedure, and the number or proportion of patients for whom this was required). Modification outcomes also included changes to any accompanying intervention related to the innovative procedure. For example, one study^42^ exploring a new method of transvesical suprapubic externalization of ureteral stents reported that modification from local anaesthesia to midazolam sedation was required. Modifications also included changes to patient selection during the course of the study.

Modifications were reported in 24 studies. The number of studies reporting modifications was broadly in line with the IDEAL framework, the majority being IDEAL stage 2a (innovations in early development). Most modifications were reported using narrative text alone (11 studies) and less frequently using flow diagrams, figures or graphs.

#### Unanticipated events (advantages and disadvantages)

Outcomes that related to an unexpected advantage or disadvantage were categorized as unanticipated events. These were further defined as occurring during or after the procedure. At least one unanticipated event was reported in eight of 42 studies (excluding protocols). These were mostly disadvantages, such as clinical complications (for example, peritonitis due to spontaneous perforation of a sigmoid diverticulum in a study of robot‐assisted anterior partial prostatectomy^70^). In one study^34^, an unanticipated event was reported as advantageous by serving as a useful safety check for the procedure.

#### Surgeon/operator experience of innovative procedure/device

Some 65 outcomes (3·7 per cent) were categorized as relating to surgeons' experiences of performing the innovative procedure or experiences of operators using the new device (if not a surgeon). Examples included ergonomics, such as pain or discomfort when carrying out the procedure, or surgeons' reports of how difficult or easy it was to perform the procedure. Further outcomes categorized under this domain included descriptions of problematic points in undertaking the procedure and impressions of the learning experience. Although 23 studies included outcomes relating to surgeon or operator experiences, only three^33^, ^45^, ^58^ reported that experiences were assessed using a formal process (including a rating scale/score, questionnaire and discussion in review meetings between cases). There was a slight trend for surgeon/operator experiences to be measured/reported in studies in the earlier stages of the innovation lifecycle (IDEAL stage 1 and 2a).

#### Patient experiences of innovative procedure/device

Overall, 215 outcomes (12·4 per cent) were categorized as assessing patients' experiences. The majority (200, 11·5 per cent) were measured after operation and were similar to those measured in effectiveness studies, such as patient‐reported quality of life and pain. A minority of outcomes (15, 0·9 per cent; in 6 different studies), however, were specifically relevant to the experiences of undergoing the innovative procedure or receiving treatment with the innovative device. One study^39^ of percutaneous nephrolithotomy under local anaesthesia, for example, reported patients' experiences specific to undergoing the innovative procedure itself, made possible because the procedure did not require the patient to be under general anaesthetic. Outcomes included patient ‘disquietness’ (restlessness or uneasiness) and good perioperative cooperation. Further examples of patients' experiences specific to undergoing the innovative procedure or receiving treatment with the innovative device related to psychological or emotional experiences. Details of how these outcomes were measured, however, were lacking. Most studies reporting innovation‐specific patient experience outcomes were reported in IDEAL stage 2a (*Table* *4*).

#### Resource use specific to innovative procedure/device

A small number of outcomes (26, 1·5 per cent) measured resource use specific to the innovative procedure/device. Examples included the cost of the procedure/device or associated costs (such as equipment sterilization costs) and the number of further treatments required owing to complications that had developed as a result of the innovative procedure. Some 31 studies included any kind of outcome relating to resource use (*Table* *4*); however, the majority of outcomes were shared with those typically measured in later‐phase studies, such as duration of surgery (72 outcomes, 4·1 per cent) and duration of hospital stay (23, 1·3 per cent).

#### Other innovation‐specific outcome domains

These domains included: details of patients suitable for the procedure in future; details of operator training/expertise necessary to perform the procedure in future; and mechanical /technical problems with the device. Although few outcomes were categorized into these domains (35, 2·0 per cent), they were considered relevant outcomes important for the evaluation of innovation.

## Discussion

Although the majority of outcomes extracted reflected those traditionally measured and reported in effectiveness studies^4^, this review identified several novel innovation‐specific outcome domains that reflect the dynamic process of surgical innovation. Broadly, these encompassed outcomes relating to procedure completion success/failure, modifications (to the procedure, concomitant interventions, or patient selection), unanticipated events, and innovation‐specific surgeon/operator experience, patient experience and resource use. Although it is recognized that unanticipated events may also occur and are reported in effectiveness studies, they are generally rare and less likely to occur when the procedure has stopped evolving and has stabilized. Unanticipated events may indicate unexpected problems and, as such, be a key driver for modifying the procedure/device, particularly if they are related to patient safety. Unanticipated events were, therefore, considered to be of key importance for evaluating innovation and conceptualized as an innovation‐specific domain. These findings served as one of multiple data sources to inform a Delphi survey and consensus study to develop a COS for early‐phase studies of surgical procedures and devices^8^.

The heterogeneity observed in the number of studies measuring and reporting outcomes of specific relevance to evaluating innovation highlights the need for more detailed guidance and improved standardization of outcomes that are important to measure when evaluating surgical innovation. The IDEAL framework provides clear guidance regarding the characteristics of studies at each stage of evaluation of surgical innovation, but uses only broad terms to describe the types of outcome that should be measured at different stages of evaluation. The lack of specific guidance around the selection and measurement of recommended outcomes may be one reason why the widespread uptake of IDEAL has been relatively limited^18^, ^72^.

The review was conducted using robust and established methodology, but has limitations. Methods to identify IDEAL/IDEAL‐D studies selected publications that had included IDEAL as a text word in the title or abstract. This pragmatic filtering step to identify relevant studies was based on the rationale that studies firmly engaging with the IDEAL framework, and therefore most likely to have more thoroughly considered outcomes in the context of evaluating innovation, may be more likely to describe their study as an IDEAL study in the title or abstract. It is accepted that some studies undertaken within the IDEAL framework may have been missed as a result of this search strategy. The number of studies identified (48) may be considered small given that the IDEAL framework was published 10 years ago. This number is, however, consistent with a recent review^18^ examining the uptake and use of the IDEAL framework. The high proportion of studies published in recent years (2017–2018) suggests a recent increase in uptake of the IDEAL framework^18^. It is acknowledged that publications regarding themselves as IDEAL studies may not necessarily have followed the IDEAL framework from the outset. Authors may have been asked to report the IDEAL stage in their reports by journal editors and reviewers during the publication process. The selection and measurement of outcomes may, therefore, have been chosen independently of the IDEAL framework and recommendations. Analysis of data relied on author‐reported information, which may not have always been precise. For example, comparison of studies across the IDEAL stages relied on the author‐reported classification of IDEAL stage. It is recognized that classification of studies can be complex and author reports of IDEAL stage may not always be accurate^18^. Similarly, categorization of outcomes as unanticipated (rather than anticipated) also relied on author‐reported information. If authors did not specifically report that an outcome was unanticipated, it was categorized as anticipated. It is possible, therefore, that the number of unanticipated events in the studies may have been higher. A final limitation is that individual outcomes were categorized into a single domain. In some instances, an outcome could have been included in more than one domain; for example, the overall desired effect of the procedure may have been measured by a patient‐reported outcome. Reporting of the number of outcomes within the different outcome domains was, therefore, dependent on the judgement of the reviewers involved in the outcome categorization/checking process. Although exact reporting of the number of outcomes relevant to each domain was not a key objective in the present study, future studies where this is important could follow the methodology recommended for classifying outcomes from effectiveness studies and classify outcomes into multiple relevant domains^4^.

The identification of innovation‐specific outcome domains is an important first step in developing a generic COS to standardize outcome selection, measurement and reporting in studies of novel surgical procedures. Such a COS would be appropriate for use before definitive evaluation within an RCT and is intended to support standardized evaluation of surgical procedures and medical devices throughout the stages of the innovation lifecycle, to facilitate safe and transparent introduction. The present review is one of several data sources to inform a conceptual framework for outcome domains for the COS to ensure its validity and comprehensiveness^8^. Given the diversity of procedures that could be performed, the feasibility of developing a generic COS for all innovative procedures could be questioned. The innovation‐specific domains identified in this review, however, are sufficiently broad that they could be applied across a wide range of procedures and devices. An international consensus study involving a diverse range of key stakeholders to agree a final core set of domains for early‐phase studies has now been completed^8^. This includes the development of clear reporting guidelines to ensure that the COS is adopted and reported in a meaningful way. A further challenge will be to determine how best to measure these key outcome domains. Engagement and creation of appropriate resources with key stakeholders, including surgeons from various disciplines, will be required to optimize the uptake and value of these tools.

## Supporting information

**Appendix S1:** supporting information
