# Supplementary material for: Outcome selection, measurement and reporting for new surgical procedures and devices: a systematic review of IDEAL/IDEAL‐D studies to inform development of a core outcome set
Source: BJS Open. 2020 Oct 4;4(6):1072–83. doi: 10.1002/bjs5.50358 (PMC8444278; doi:10.1002/bjs5.50358)
Supplement: Supplementary file 1 — Appendix S1: supporting information [file BJS5-4-1072-s001.docx]

**BJS5_­50358**

**Outcome selection, measurement and reporting for new surgical procedures and devices: a systematic review of IDEAL/IDEAL-D studies to inform development of a core outcome set**

R. C. Macefield, N. Wilson, C. Hoffmann, J. M. Blazeby, A. G. K. McNair, K. N. L. Avery and S. Potter

**Table S1 Categorisation of outcomes reported in included studies, by IDEAL stage (n=48 studies)**

|  |  | **Number of outcomes per IDEAL stage, n (%)** | | | | | | TOTAL |
| --- | --- | --- | --- | --- | --- | --- | --- | --- |
|  | **IDEAL stage** | 1 | 2a | 2b | 3 | Multi-stage | Not stated |  |
| **Outcome domains** **specific to innovation** | Procedure completion success/failure | 31 (7.40) | 40 (6.94) | 11 (3.32) | 1 (5.88) | 20 (6.37) | 4 (5.00) | 107 (6.16) |
|  | Modification: to procedure | 28 (6.68) | 33 (5.73) | 7 (2.11) | 0 | 20 (6.37) | 0 | 88 (5.07) |
|  | Modification: to concomitant intervention | 0 | 1 (0.17) | 0 | 0 | 0 | 0 | 1 (0.06) |
|  | Modification: to patient selection during study | 1 (0.24) | 0 | 0 | 0 | 2 (0.64) | 0 | 3 (0.17) |
|  | Unanticipated advantages* | 1 (0.24) | 0 | 0 | 0 | 0 | 0 | 1 (0.06) |
|  | Unanticipated disadvantages* | 1 (0.24) | 7 (1.22) | 0 | 0 | 3 (0.96) | 0 | 11 (0.63) |
|  | Surgeon/ operator's experience | 19 (4.53) | 13 (2.26) | 4 (1.21) | 0 | 28 (8.92) | 1 (1.25) | 65 (3.74) |
|  | Patients' experience of the innovative procedure/device* | 1 (0.24) | 4 (0.69) | 8 (2.42) | 0 | 0 | 2 (2.50) | 15 (0.86) |
|  | Required resource use specific to the innovative procedure/device* | 7 (1.67) | 4 (0.69) | 8 (2.42) | 0 | 4 (1.27) | 3 (3.75) | 26 (1.50) |
|  | Details of patients suitable for procedure in future | 0 | 4 (0.69) | 2 (0.60) | 0 | 0 | 0 | 6 (0.35) |
|  | Details of operator training/expertise necessary to perform procedure in future | 0 | 1 (0.17) | 1 (0.30) | 0 | 9 (2.87) | 0 | 11 (0.63) |
|  | Mechanical/technical problems with device, if applicable | 0 | 2 (0.35) | 8 (2.42) | 1 (5.88) | 7 (2.23) | 0 | 18 (1.04) |
| **Outcome domains shared with effectiveness studies** | Overall desired effect of procedure /device achieved | 48 (11.46) | 118 (20.49) | 29 (8.76) | 0 | 35 (11.15) | 24 (30.00) | 254 (14.62) |
|  | Anticipated advantages* | 19 (4.53) | 17 (2.95) | 10 (3.02) | 1 (5.88) | 6 (1.91) | 0 | 53 (3.05) |
|  | Anticipated disadvantages* | 110 (26.25) | 97 (16.84) | 142 (42.90) | 6 (35.29) | 84 (26.75) | 34 (42.50) | 473 (27.23) |
|  | Duration of procedure | 27 (6.44) | 15 (2.60) | 7 (2.11) | 0 | 23 (7.32) | 0 | 72 (4.15) |
|  | Duration of hospital stay | 7 (1.67) | 7 (1.22) | 6 (1.81) | 0 | 3 (0.96) | 0 | 23 (1.32) |
|  | Patient’s physical/psychological experiences after the procedure | 36 (8.59) | 79 (13.72) | 41 (12.39) | 6 (35.29) | 34 (10.83) | 4 (5.0) | 200 (11.51) |
| **Other** | Common data elements | 58 (13.84) | 107 (18.58) | 30 (9.06) | 0 | 22 (7.01) | 6 (7.50) | 223 (12.84) |
|  | Too broad for categorisation (e.g. “effectiveness”) | 25 (5.97) | 27 (4.69) | 17 (5.14) | 2 (11.76) | 14 (4.46) | 2 (2.50) | 87 (5.01) |
| **Total number of outcomes, all studies** | | **n=419** | **n=576** | **n=331** | **n=17** | **n=314** | **n=80** | **n=1737** |

*combined to include during or after the procedure
